# Artificial Intelligence in Hypertension Management: An Ace up Your Sleeve

**DOI:** 10.3390/jcdd10020074

**Published:** 2023-02-09

**Authors:** Valeria Visco, Carmine Izzo, Costantino Mancusi, Antonella Rispoli, Michele Tedeschi, Nicola Virtuoso, Angelo Giano, Renato Gioia, Americo Melfi, Bianca Serio, Maria Rosaria Rusciano, Paola Di Pietro, Alessia Bramanti, Gennaro Galasso, Gianni D’Angelo, Albino Carrizzo, Carmine Vecchione, Michele Ciccarelli

**Affiliations:** 1 Department of Medicine, Surgery and Dentistry, University of Salerno, 84081 Baronissi, Italy; 2 Department of Advanced Biomedical Sciences, Federico II University of Naples, 80138 Naples, Italy; 3 Cardiology Unit, University Hospital “San Giovanni di Dio e Ruggi d’Aragona”, 84131 Salerno, Italy; 4 Hematology and Transplant Center, University Hospital “San Giovanni di Dio e Ruggi d’Aragona”, 84131 Salerno, Italy; 5 Department of Computer Science, University of Salerno, 84084 Fisciano, Italy; 6 Vascular Physiopathology Unit, IRCCS Neuromed, 86077 Pozzilli, Italy

**Keywords:** hypertension, artificial intelligence, machine learning, blood pressure, deep learning, deep neural networks, big data, wearable technology, digital health, photoplethysmograph

## Abstract

Arterial hypertension (AH) is a progressive issue that grows in importance with the increased average age of the world population. The potential role of artificial intelligence (AI) in its prevention and treatment is firmly recognized. Indeed, AI application allows personalized medicine and tailored treatment for each patient. Specifically, this article reviews the benefits of AI in AH management, pointing out diagnostic and therapeutic improvements without ignoring the limitations of this innovative scientific approach. Consequently, we conducted a detailed search on AI applications in AH: the articles (quantitative and qualitative) reviewed in this paper were obtained by searching journal databases such as PubMed and subject-specific professional websites, including Google Scholar. The search terms included artificial intelligence, artificial neural network, deep learning, machine learning, big data, arterial hypertension, blood pressure, blood pressure measurement, cardiovascular disease, and personalized medicine. Specifically, AI-based systems could help continuously monitor BP using wearable technologies; in particular, BP can be estimated from a photoplethysmograph (PPG) signal obtained from a smartphone or a smartwatch using DL. Furthermore, thanks to ML algorithms, it is possible to identify new hypertension genes for the early diagnosis of AH and the prevention of complications. Moreover, integrating AI with omics-based technologies will lead to the definition of the trajectory of the hypertensive patient and the use of the most appropriate drug. However, AI is not free from technical issues and biases, such as over/underfitting, the “black-box” nature of many ML algorithms, and patient data privacy. In conclusion, AI-based systems will change clinical practice for AH by identifying patient trajectories for new, personalized care plans and predicting patients’ risks and necessary therapy adjustments due to changes in disease progression and/or therapy response.

## 1. Introduction

Arterial hypertension (AH) is a global public health problem, and its treatment is primarily aimed at reducing associated cardiovascular (CV) morbidity and mortality [1,2]. AH affected more than 1.13 billion individuals in 2015, and the prevalence appears to affect approximately 35–45% of Campo’s overall population [3]. Moreover, AH is the most significant contributor to the global burden of CV diseases and represents a heavy socio-economic burden for many countries [4,5]; indeed, even moderate elevations in arterial blood pressure (BP) are associated with a significant reduction in life expectancy [6]. Furthermore, current data suggest that over 14 million people are unaware of their abnormal BP level; consequently, they are not receiving appropriate medication for it, nor do they engage in other interventions to maintain BP in the normal range [7]. Regardless of guidelines, BP control in hypertensive patients in treatment is insufficient due to many factors, including poor adherence to therapy [8]. Moreover, the definition of “hypertension” recapitulates several different sub-phenotypes influenced by multiple variables: gender, BMI, lifestyle conditions, and so on [9]. Furthermore, the pharmacological therapy used to treat essential AH has remained substantially unchanged in the last 20 years and is mainly focused on regulating vascular resistance [1,10]. Therefore, there is an evident gap in the knowledge required to deepen the multifaced aspects of AH and prompt the research and development of novel approaches.

In this scenario, the possibility of collecting, storing, and analyzing multiple pieces of information from a single patient in the form of electronic health records (EHRs) requires revising the conventional healthcare model of AH management using new technologies or monitoring techniques [11,12]. In this context, artificial intelligence (AI) is a technological method that has been in development in recent years [13,14,15], and, if used appropriately, it could have surprising results in developing predictive models of AH, formulating the diagnosis, stratifying patients, and identifying the most effective therapy (Table 1).

Specifically, accurate BP estimation is essential because BP is a risk factor for many clinical CV events, including stroke and dementia, and therefore can be integrated into several models for risk stratification [16,17]; moreover, in the computational simulation of CV disease, BP can influence the value of focal hemodynamic metrics, e.g., fractional flow [18]; consequently, patient-specific BP values can improve the accuracy of simulation results and have been applied in some recent models [19,20].

Therefore, AI-based systems might change clinical practice for AH by identifying patient trajectories for new, personalized care plans and predicting patients’ risks and necessary therapy adjustments due to changes in disease progression and/or therapy response. Accordingly, a basic knowledge of this science is essential because AI might change medical practice jobs: tiring and routine tasks could be completed by computers to free up time and allow cardiologists to carry out more difficult and sensitive tasks. Therefore, in this review, we describe multiple applications of AI, encompassing diagnostic, prognostic, and therapeutic issues currently unsolved in managing AH (Tables S1–S4).

Overall, this manuscript aims to provide a complete picture of the state of the art of AI in AH (primary and secondary) management, analyzing every aspect of the diagnosis, treatment, and patient follow-up, without neglecting the limitations and all of the possible tools to overcome them.

## 2. The Principles of AI

**Figure 1:**
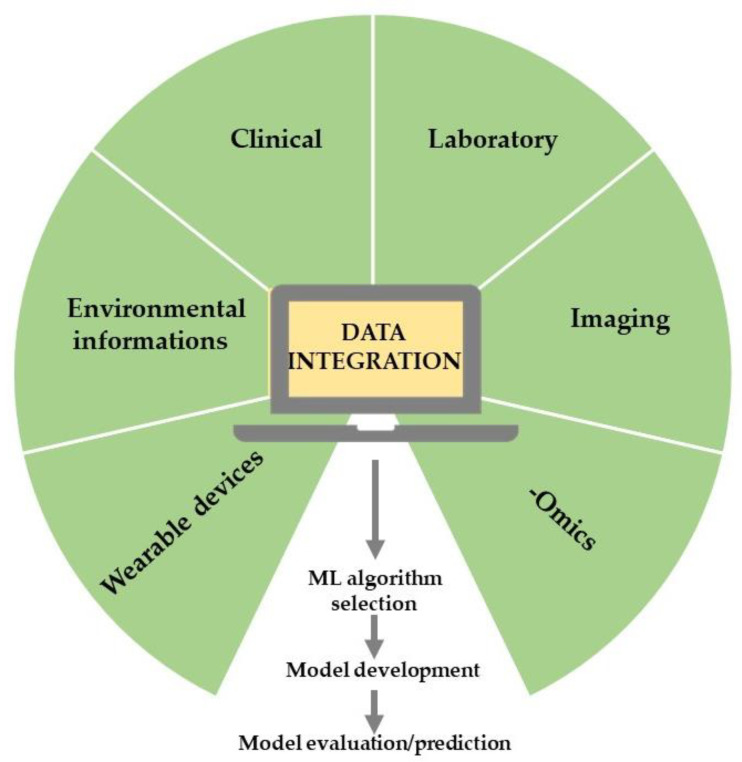
The typical ML workflow in healthcare research.

AI is a wide-ranging branch of computer science concerned with building smart machines capable of increasing their knowledge through an automatic learning process that typically requires human intelligence [21,22,23]. Therefore, AI is an interdisciplinary science with multiple approaches that incorporate reasoning (making inferences using data), natural language processing (ability to read and understand human languages), planning (ability to act autonomously and flexibly to create a sequence of actions to achieve a final goal), and machine learning (ML) (algorithms that develop automatically through experience) [21]. Specifically, AI based on ML techniques [15] is used to perform predictive analyses by examining mechanisms and associations among given variables from training datasets, which may consist of a variety of data inputs, including wearable devices, multi-omics, and standardized EHRs [10,24]. Essentially, in ML, the rules would be learned by algorithms directly from a set of data rather than being encoded by hand [25]; consequently, by using specific algorithms, ML can establish complex relationships among data, rules governing a system, behavioral patterns, and classification schemes [15]. The classic ML process begins with data acquisition, continues with feature extraction, algorithm selection, and model development, and leads to model evaluation and application [26] (Figure 1). Supervised and unsupervised learning are the most popular approaches employed in ML. Supervised learning is used to predict unknown outputs from a known labeled dataset, hypotheses, and appropriate algorithms, such as an artificial neural network (ANN), support vector machine (SVM), and K-nearest neighbor. The choice of the technique depends on the dataset’s features, number of variables, learning curve, training, and computation time [27,28]. Specifically, supervised learning provides predictions from big data analytics but requires manually labeled datasets and biases that can arise from the dataset itself or the algorithms [24].

On the other hand, in unsupervised learning techniques, there is no information on the features to be predicted; consequently, these techniques must learn from the relationships among the elements of a dataset and classify them without basing them on categories or labels [22]. Therefore, they look for structures, patterns, or characteristics in the source data that can be reproduced in new datasets [15]. ML mainly mimics the nervous system’s structure by creating ANNs, which are networks of units called artificial neurons structured into layers [29]. The system learns to generate patterns from data entered in the training session [29]. A specific ANN, consisting of more layers that allow for improved predictions from data, is known as a deep neural network (DNN). Its performance could be enhanced as the dimension of the training dataset rises [25]. Still, it largely depends on the distribution gap between training and test datasets: a highly divergent test dataset would test an ML prediction model on a feature space that it was not trained on, resulting in poor testing and results; additionally, a highly overlapping test dataset would not test the model for its generalization ability [30]. Specifically, DL employs algorithms such as DNNs and convolutional neural networks (CNNs) [15]. Nevertheless, regardless of its capability of using unlabeled datasets, unsupervised learning still has some limitations, such as the generalizability of cluster patterns identified from a cohort of patients, which can lead to overfitting to the training dataset, and the need to be validated in different large datasets [24]. In the real world, AI can provide tools to improve and extend the effectiveness of clinical interventions [15]. For example, incorporating AI into hypertension management could improve every stage of patient care, from diagnosis to therapy; consequently, the clinical practice could become more efficient and effective.

## 3. Methods

We conducted a detailed search on AI applications in AH. The research includes topics ranging from big data to complex technology-based interventions. Specifically, the articles reviewed in this paper were obtained by searching journal databases such as PubMed and subject-specific professional websites, including Google Scholar. The search terms included artificial intelligence, artificial neural network, deep learning, machine learning, big data, arterial hypertension, blood pressure, blood pressure measurement, CV disease, and personalized medicine. The inclusion criteria focus on articles directly or indirectly related to the topic of AI and AH. Specifically, both quantitative (measurable data) and qualitative (reasons, opinions, and motivations) reports were reviewed.

The authors independently screened titles and abstracts; subsequently, full texts were sourced for relevant articles. The reference lists of included trials and meta-analyses were also reviewed for significant articles.

## 4. AI in the Measurement of Blood Pressure

The commonly used methods for BP monitoring are either non-invasive inflatable cuff-based oscillometric or invasive arterial line manometric measurement. The former takes intermittent measures because a pause of at least 1–2 min between two BP measurements is necessary to avoid errors in the measurement [31,32]; moreover, the inflation of the cuff may disturb the patient, and the consequences of these disturbances are alterations in BP [33]. On the other hand, invasive arterial line manometric measurement has an elevated risk of complications; consequently, these unsolved issues drive the search for new non-invasive BP monitoring techniques.

**Figure 2:**
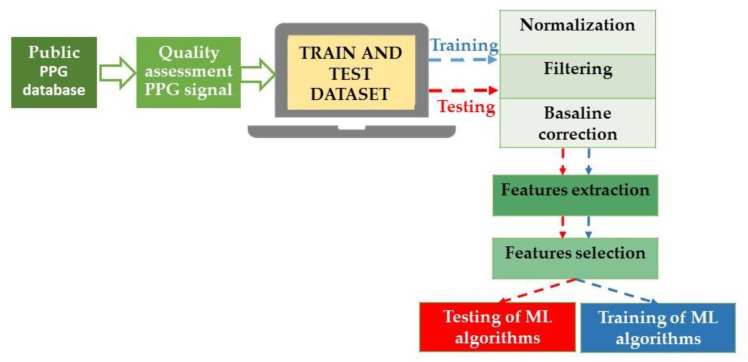
Block diagram of the blood pressure estimation process using ML techniques. In detail, the raw signals are prepared through normalization, the correction of baseline wandering due to respiration, and finally, signal filtration. Specifically, to construct a dataset for BP estimation models, it is necessary to accurately extract the features of the original waveform (and underlying demographic and statistical data) and select effective features, improving the generalization and reducing the risk of overfitting the algorithms. PPG: photoplethysmograph; ML: machine learning.

In this scenario, AI algorithms could help improve precision, accuracy, and reproducibility in diagnosing and managing AH using emerging wearable technologies. Alternatives for monitoring BP are cuff-based devices (such as volume-clamp devices or wrist-worn inflatable cuffs) and cuffless devices that use mechanical and optical sensors to determine features of the blood pulse waveform shape (for example, tonometry [34], photoplethysmography [35], and capacitance [32]). In particular, cuffless blood pressure monitoring has been evaluated using a two-step algorithm with a single-channel photoplethysmograph (PPG). This system achieved an AAMI/ISO standard accuracy in all blood pressure categories except systolic hypotension [36]. Independently of the acquisition method, the received signals are preprocessed and sent for feature extraction and selection. Subsequently, the signals and the gathered data can be used to feed ML to obtain systolic BP (SBP) and diastolic BP (DBP) estimations from the raw signals [35] (Figure 2).

Since the volume and distension of arteries can be related to the pressure in the arteries, the PPG signal produces pulse waveforms that are similar to pressure waveforms created by tonometry. PPG offers the added advantage that it can be measured continuously using miniature, inexpensive, and wearable optical electronics [37]. However, PPG signal measurements are not without technical challenges; indeed, they require noise elimination, multisite measurement, multiphotodector development, event detection, event visualization, different models, the accurate positioning of sensors, and the calculation of propagation distances, without neglecting the impact of the variable PEP time on the pulse wave velocity timing [37]. Moreover, there are several PPG-based methods for estimating BP: the PPG signal alone and its derivate, ECG and PPG signals, BCG and PPG signals, and PCG and PPG signals; each has advantages and limitations [38,39,40,41,42], which, however, are beyond this discussion.

### ML Algorithms in BP Estimation

To adapt to the nonlinearity of the dataset and to create a relationship between features and estimated BP, there are different ML approaches [43]:

1. Gaussian Process Regression: A Bayesian regression approach gives a probability distribution over all possible values [35,44].
2. Ensemble trees: The idea is to pull together a set of weak learners to create a strong learner [45].
3. Multivariate Linear Regression: It is a method to analyze the correlation, correlation direction, and strength between multiple independent variables and the dependent variable [33,46,47].
4. Support vector regression: It is a non-parametric algorithm that uses a kernel function (a class of algorithms for pattern analysis, whose general task is to find and study relations in datasets) [48,49,50,51].
5. Random forest, gradient boosting, and adaptive boosting regression [48,49].
6. CNN [52,53].

After hyper-parameter optimization, it is necessary to evaluate the performance of ML algorithms through the correlation between the acquired predicted data and the ground-truth data. The difference between reference and estimated BP could be considered using the following criteria: the mean absolute error, mean squared error, and correlation coefficient [35]. The role of parameter optimization is to lower the value of the predicted error. The mean absolute and standard deviations are the model’s predictive performance indicators.

Specifically, these AI-based systems could help continuously monitor BP using wearable technologies and improve AH management and outcomes [33,50]. Starting from the input (raw signals), we can reach the output (estimated SBP and DBP) through the algorithms of ML [24]. In particular, BP can be estimated from a PPG signal obtained from a smartphone or a smartwatch by using DL [54,55].

Moreover, future studies on AI and wearable devices need to confirm the above results and provide conclusive clinical data to support using a combination of AI and wearable-device-obtained data to correctly perform BP measurements, which may offer an alternative to current oscillometric methods [24].

## 5. Use of AI for the Prediction of Undiagnosed Hypertension

AH is generally asymptomatic or variably occurs with symptoms such as headache, dizziness, tinnitus, and nosebleeds in a minority of patients. For this reason, it is not always possible to readily identify and predict this disease.

The goal should be to implement primary and secondary prevention methods. This task could greatly benefit AI implementation, featuring the individual identification of each patient’s previous and new CV risk factors and treating modifiable risk factors. AI can predict the patient’s risk of AH development thanks to different algorithm settings with big sets of data. Predicting AH onset has been attempted in the last decade with progressively greater precision thanks to technological advancements.

Accordingly, risk factors for AH, such as low levels of education, a sedentary job, a family history of AH, demographic data, routine blood tests, BMI, waist and hip circumference and ratio, diet, physical exercise, and salt and alcohol intake, showed effectiveness in predicting elevated SBP using ML techniques [56,57,58], for instance, an ANN [59]. On the other hand, AI techniques are also emerging in gene expression analysis; specifically, it is possible to improve AH predictive models by integrating data from a combination of gene expression and next-generation sequencing in ML analysis [60]. Furthermore, thanks to ML algorithms, it is possible to identify new hypertension genes; accordingly, Li et al. [61] analyzed gene expression in hypertensive patients, thus identifying 177 new hypertension genes thanks to ML algorithm development: this was then integrated with environmental factors in evaluating the risk of AH [53,62].

Furthermore, health risk prediction using a DL architecture appears suitable for extensive complex datasets, as shown by Maxwell et al. [63]. Using physical examination records of 110,300 patients, it was possible to examine, classify, and learn each risk factor contributing to chronic diseases such as AH, diabetes, and liver steatosis [63].

With the same principle, Ye et al. [56] validated an accurate 1-year risk prediction model for the incidence of essential AH. In this retrospective and prospective study, risk factors (type 2 diabetes, dyslipidemias, CV diseases, etc.) were calculated, evaluated, and stratified, yielding an accuracy of 91.7% for the prediction of the AH incidence at 1 year, with 87% in the prospective cohort (validation) [56].

Furthermore, the AI risk assessment of AH development can also benefit from cardiorespiratory fitness data obtained from treadmill exercise stress testing [64]. Overall, AI has the potential to increase efficacy by aggregating immense volumes of information, virtually predicting the risk of AH and consequently preventing or delaying the development of this disease by directing early interventions [24].

Making the AH diagnosis using validated guidelines [65,66] is an easy task for AI. Using inputs such as BP, comorbidities, demographic data, and routine blood tests, the accuracy can reach 92.85% with an ANN [67]. With a similar dataset (age, BP, BMI, serum lipoproteins profile, smoking habit, and exercise), using ML, the accuracy is estimated at 82% [68].

The most common example of AI for AH diagnosis, and probably still the most accurate, is twenty-four-hour ambulatory BP monitoring (ABPM) [69]. Thanks to direct and regular interval BP measurements and a relatively easy algorithm, ABPM software can identify and classify AH. ABPM is particularly useful in correctly evaluating white coats and masked AH [70,71].

New approaches based on ANNs try to estimate BP for AH diagnosis in healthy individuals using factors such as BMI, age, exercise, smoking, and alcohol consumption, although with limited accuracy and efficacy [68]. An interesting method for SBP prediction is the use of retinal fundus images. This factor was used by Poplin et al. [72], who showed that DL could be used in this case, even if the BP error was 11.23 mmHg, clearly not accurate enough to be of diagnostic use.

The ability of AI to diagnose AH is dependent on its accuracy. Further studies will identify more methods of BP prediction for the early diagnosis of AH and complication prevention. Looking at the world around us, AI’s future in AH diagnosis and management probably lies in systems that can constantly retrieve vital signs, such as smartphones, smartwatches, and all of the relative accessories [73].

## 6. Targeting AH by AI

Optimizing pharmacological therapy to achieve the optimal control of BP levels represents a tough challenge for physicians. The management of AH is based on evidence-based medicine and expert opinions; BP control rates remain poor worldwide and are far from satisfactory across Europe [74].

Specifically, the importance of AI in this field is in the robust identification of modifiable factors that impact the evolution of AH; moreover, this new instrument could support the choice of the most appropriate management of AH. Indeed, most hypertensive patients need medication and lifestyle changes to achieve optimal BP control, and AI is helpful due to its better capability of identifying the best combination therapy compared to standard analysis [74].

Several works discussed and analyzed the opportunity of using AI to evaluate the impacts of different factors on BP values. Koren et al. applied ANN and decision trees to identify parameters contributing to Campo’s success in his AH drug treatment [75]. Indeed, ML algorithms allow better real-world data analysis, identifying patterns and trends not easily recognized with classical experimental or observational approaches. Randomized trials usually compare the effectiveness of a drug to a placebo or, at best, to an already-approved compound. However, evaluating the differences between and advantages of drug combinations, frequently necessary for AH treatment, is a more complex task [75] that AI could fulfill. Here, we provide some previous experience and approaches; for example, the computer was presented with a training dataset in the first training phase, and for each instance, it was given the correct classification. Specifically, the appropriate classification of the algorithm was divided into two reference categories: “treatment success”, defined as BP lower than 140/90 mmHg within 90 days after starting therapy, and “treatment failure” in any other case. Finally, AI decision trees and ANN compared various classes of antihypertensives in patients: beta-blockers alone or in combination were the most effective [75]. In another study, ML methods predicted individual treatment effects of intensive BP therapy [76]; notably, the results revealed an improvement in the discrimination and calibration of individualized medications from clinical trial data. Moreover, Ye X. et al. [77] demonstrated the potential of using predictive models to select optimal AH treatment pathways; specifically, along with clinical guidelines and guideline-based CDS systems, the LSTM models show the best prediction for achieving the optimal control of BP with different combinations of treatments. ML can also detect adherence to antihypertensive therapy by analyzing data recorded from a smartphone application to promote patients’ awareness, self-monitoring, and treatment compliance [78].

Nonetheless, the exploitation of AI in the healthcare field is still in its early phases, and its potential is not entirely explored. Indeed, AI represents the opportunity for a global evaluation of the patient in his or her complexity because it allows the integration of clinical, demographic, biochemical, and instrumental data to develop models that physicians and healthcare providers could use to improve AH management and test new drug therapies using the multi-omics method [79,80].

We are now entering an era in which omics-based technology evolution will start to deliver long-promised elements that may improve the understanding of the complex mechanistic basis of this disease [79]. Accordingly, the key to better disease management will pass through personalized medicine. The key to this is future drug discovery that will be possible thanks to enhanced AI technologies exploiting information integration. Data from sequenced genomes, functional genomics, protein profiling, metabolomics, and bioinformatics may ensure a better comprehensive systems-based analysis for further understanding AH disease’s complexities. Integrating AI with omics-based technologies will lead to the definition of the trajectory of the hypertensive patient and the use of the most appropriate drug.

In conclusion, AI can improve intelligent healthcare systems that provide personalized recommendations and treatment approaches [24].

## 7. Definition of the Hypertensive Patient’s Trajectory: Role of AI in AH Prognosis

According to the recent AHA and ESC guidelines [74,81], the evaluation of the prognosis of AH is related to the demographic characteristics, the progression of organ injury, and comorbidities of the patients. Predicting the outcomes of hypertensive patients is significant research work [82].

There are currently several clinical risk scores intended for specific populations and risk groups [83,84,85]. Nevertheless, most of these scores are based on linear models and might therefore lack specificity and sensitivity in certain subgroups [86]. Moreover, the wide variety of scores may also cause slow adoption in real-world clinical practice [86].

The role of AI concerning the prognostic establishment of AH is due to the utility of ML algorithms trained on large datasets to estimate prognoses and potentially guide medical treatments [87].

The recent focus on AI and ML methods for AH prognosis is related to the critical repercussions of elevated BP for the risk of developing organ damage. Despite commonly used statistical methods, AI systems can process large amounts of complex data. Therefore, AI and ML aim to process a prognostic model that has clinical relevance in managing patients in the real world [88]. The prognostic impact of AH needs the stratification of patients based on their global evaluation with the integration of different parameters, such as the grade and stage of AH, BP control, and concomitant comorbidities. Indeed, AI can stratify patients correctly through the use of classification algorithms (SVM, C4.5 decision tree, random forest (RF), and extreme gradient boosting (XGBoost)); specifically, XGBoost has the best prediction performance [82].

However, the accuracy of the definition of the individual trajectory can be enhanced by a prolonged follow-up [89] and an increase in the number of relevant features, such as genomic variants associated with specific hypertensive phenotypes and different outcomes [61,90].

Moreover, the prognosis determines the frequency and the type of clinical monitoring. Specifically, AI allows the elaboration of new risk stratification to develop novel prognostic layers in favor of personalized medicine with the prospect of efficiently measuring the grade classification of AH [91] and identifying different outcomes’ profiles [89]. In conclusion, the clinical advantages of implementing AI systems and their integration into current prognostic stratification might grant the correct identification of classes of outcomes of patients, improving their clinical management.

## 8. AI in Secondary Arterial Hypertension

The causes of hypertension are multiple, and we must not overlook secondary arterial hypertension; indeed, in 10–15% of cases, the specific cause underlying hypertension can be identified [92]. Specifically, to arrive at the diagnosis of essential hypertension, all causes of secondary hypertension must be ruled out, and the accurate history, thorough examination, and performance of all necessary tests based on the data collected always place the physician and his or her knowledge of internal medicine at the basis of the evaluation of each hypertensive patient. Consequently, diagnosis and patient management must be connected to the clinician, who can use AI to speed up and facilitate his or her task. In any case, AI and ML cannot wholly replace the doctor’s role, but they represent a useful tool in his hands. Identifying secondary hypertension in its various subtypes is essential to preventing and targeting the treatment of CV complications. However, screening for secondary hypertension can be time-consuming, expensive, and difficult; consequently, simplified diagnostic tests are urgently required to distinguish between primary and secondary hypertension to address the current underdiagnosis of the latter. In children and adolescents, the most common causes of hypertension are renal parenchymal or vascular disease and aortic coarctation [93]; in adults, earlier studies identified renal parenchymal and vascular diseases as the most common causes of secondary hypertension. Obstructive sleep apnea (OSA) was recognized as an exceedingly common cause of secondary hypertension [94]. Among endocrine causes, we include primary aldosteronism (the most common), thyroid disease (hypo- or hyperthyroidism), hypercortisolism (Cushing’s), and finally, phaeochromocytoma [92]. The application of ML methods to the etiological diagnosis of secondary hypertension can be helpful in clinical practice. Accordingly, AI technology should be implemented cautiously; to be a partner of clinicians, there is still a long way to go, but it can serve as a virtual assistant and enable clinicians to promote quality and increase efficiency. Based on EMRs from Fuwai Hospital, five ML prediction models with good performance and applicability to the etiology detection of secondary hypertension were developed by Campo [95], which demonstrated that ML approaches were feasible and effective in diagnosing secondary hypertension. Reel and colleagues [95] showed that the MOmics approach provided better discriminatory power compared to single-omics (monoomics) data analysis and appropriately classified different forms of endocrine hypertension with high sensitivity and specificity, providing potential diagnostic biomarker combinations for diagnosing secondary hypertension subtypes. However, there still needs to be more data in the literature on the application of AI in the field of secondary hypertension; consequently, these innovative and clinically relevant prediction models still require further validation and more clinical tests before being implemented into clinical practice.

## 9. Limitations of Applying ML in CV Research

Despite the great importance that AI has taken on in the last few years, it is necessary to underline that this system is not free from technical issues and biases (Table S5). The quality of the algorithms underlying AI technology can be affected by some limitations, such as the inconsistent quality of the studies that form the databases at the heart of AI [96].

Specifically, overfitting is a significant issue common to all ML models: this happens when a model has become overly attuned to the training data, such that it does not generalize to new datasets; this is the opposite of underfitting, where the algorithm cannot wholly capture the predictive power of the data. These two issues (over- and underfitting) could be solved by improving the parameters of the model or making modifications to the training set [21].

Moreover, it is also essential to consider the adversarial robustness of a model (ability to resist being fooled), which could be improved by enlarging the training set [97].

Furthermore, many clinicians remain wary of ML because of concerns about the “black-box” nature of many ML algorithms. These models are sufficiently complex that they are not directly interpretable to humans. Subsequently, the lack of interpretability of predictive models can undermine trust in those models, especially in the medical field, in which so many decisions are life-and-death issues [98]. To be trusted, users must comprehend the model outputs [98]. Consequently, the “explainability” technique in ML seeks to imbue humans with a high level of understanding of how an algorithm works and makes decisions without carefully considering each step [99]; however, the nature of explanations as approximations may omit important information about how black-box models work and why they make specific predictions [100].

Another flaw is the lack of discernment of predisposing factors for specific CV diseases from confounding factors. Moreover, it is also possible that, during data analysis processes by specialized clinicians, some may contribute to lowering the accuracy of the databases owing to their biases, thus making their validation even more time-consuming [28].

It would also be necessary for some AI-automated diagnostic CV algorithms to identify CV risk factors that are essential but still not unanimously recognized since, sometimes, there is no consensus among cardiologists. Therefore, it would be desirable to validate data through a clinical consensus before merging them into the AI, though expensive and time-consuming. Another important aspect is the disparity between various racial groups, ethnic minorities, and social classes. Some CV diseases are more represented in some races and/or ethnic minorities and can express themselves with different phenotypes [101].

Prospectively, it will be necessary to render AI accessible to patients and promote awareness, self-monitoring, healthy behaviors, and therapeutic adherence by integrating new technologies, such as wearable devices that can automatically analyze activity levels and give feedback, such as lifestyle and drug dose changes [24].

## 10. Conclusions

The study of AH needs a revolution, and its future may lie in the favorable convergence of digital data and biotechnological and biomedical sciences and their implementation in healthcare delivery with new delivery models and effective strategies for population health. With AI, we could better understand epigenetic changes relevant to AH onset and progression, potentially classify the risk of AH in individuals, identify the mechanism of poorly controlled AH, and evaluate treatment responses in clinical trials using a multi-omics approach [102]. Furthermore, AI could target healthy individuals who are at higher risk of developing AH and may benefit from lifestyle modifications for the primary prevention of CV disease [24].

In conclusion, AH prediction and management using a combination of AI and wearable technology could potentially be the first real chance for precision CV medicine [102]. Moreover, future AI-enhanced AH care will encourage patient awareness, self-monitoring, healthy behaviors, and treatment adherence, along with developing digital technologies [24]. Therefore, future research needs to focus on precision AH medicine utilizing AI-based technologies to reduce the global burden of AH without neglecting the significant limitations that this approach still has.

## Figures and Tables

**Table 1:** AI application in hypertension management.

Applications		Benefits
**Measuring BP**	Estimate BP by analyzing PPG signal with ML and DL algorithms.	Self-monitoring BP for hypertension
**Predicting AH development**	Predict the risk of developing AH by using genetics, medical data, and behavioral, environmental, and socioeconomic factors.	Timely intervention
**Diagnosing AH**	Accurately diagnosing AH by using CV risk factors, anthropometric data, vital signs, and laboratory data.	Precision diagnosis
**Predicting AH treatment success**	Identify factors contributing to treatment success.	Personalized treatment plan
**Predicting AH prognosis**	Stratify patients and predict CV outcomes.	Treatment plan adjustment

AI: artificial intelligence; BP: blood pressure; PPG: photoplethysmograph; ML: machine learning; DL: deep learning; AH: arterial hypertension; CV: cardiovascular.

## Data Availability

Not applicable.

## Supplementary Materials

The following supporting information can be downloaded at https://www.mdpi.com/article/10.3390/jcdd10020074/s1. Table S1: Major contributions of AI in AH measurement. Table S2: Major contributions of AI in AH prediction and diagnosis. Table S3: Major contributions of AI in AH treatment. Table S4: Major contributions of AI in AH outcome. Table S5: Limitations of applying ML in cardiovascular research Supplementary References [34,35,51,56,57,58,59,60,61,63,64,68,75,76,77,82,89,90,103].
