# Supplementary material for: Artificial Intelligence in Hypertension Management: An Ace up Your Sleeve
Source: J Cardiovasc Dev Dis. 2023 Feb 9;10(2):74. doi: 10.3390/jcdd10020074 (PMC9963880; doi:10.3390/jcdd10020074)
Supplement: Supplementary file 1 [file jcdd-10-00074-s001.zip › jcdd-2156148-supplementary.pdf]

Table S1. Major contributions of AI in AH measurement.

Reference (y)	Application/Aim	Methods/Population	Results/Limitations
Huang et al. (2019) PMID: 30791363 [1]	Development of a pressure-sensing array for noninvasive continuous blood pulse-wave monitoring.	Various machine-learning algorithms, including random forest regression (RFR), gradient-boosting regression (GBR), and adaptive boosting regression (ABR), were employed for estimating systolic and diastolic blood pressure from the measured pulse-wave signals.	Creation of a device for pressure-sensing array for noninvasive continuous blood pulse-wave monitoring with high sensitivity and fast dynamic response.
Chowdhury et al. (2020) PMID: 32492902 [2]	Estimating systolic and diastolic blood pressure with the help of PPG signal features and machine learning algorithms.	PPG signals were acquired from 219 subjects, which undergo preprocessing and feature extraction steps. Time, frequency, and time-frequency domain features were extracted from the PPG and their derivative signals. Feature selection techniques were used to reduce the computational complexity and to decrease the chance of over-fitting the ML algorithms. The features were then used to train and evaluate ML algorithms. The best regression models were selected for systolic BP (SBP) and diastolic BP (DBP) estimation individually.	Gaussian process regression (GPR) along with the ReliefF feature selection algorithm outperforms other algorithms in estimating SBP and DBP with a root mean square error (RMSE) of 6.74 and 3.59, respectively.
Chen et al. (2019) PMID: 31174357 [3]	Establishing the systolic BP and diastolic BP estimation models based on machine learning using pulse transit time and characteristics of pulse waveform	In the process of model construction, the mean impact value method was introduced to investigate the impact of each feature on the models and the genetic algorithm was introduced to implement parameter optimization.	The nonlinear relationship between the features and BP and had higher accuracy than the traditional methods with the error of 3.27 ± 5.52 mmHg for systolic BP and 1.16 ± 1.97 mmHg for diastolic BP. Limitations include lack of personal information and long-term monitoring.
Khalid et al. (2018) PMID: 30425819 [4]	Developing a single PPG-based cuffless BP estimation algorithm with highest accuracy in clinical and practical use	Vital sign dataset (online database) was accessed to extract raw PPG signals and its corresponding reference BPs (systolic BP and diastolic BP). The online database consisted of PPG waveforms of 32 cases from whom 8133 (good quality) signal segments (5 s for each) were extracted, preprocessed, and normalised in both width and amplitude. Three most significant pulse features (pulse area, pulse rising time, and width 25%) with their corresponding reference BPs were used to train and test three machine learning algorithms (regression tree, multiple linear regression (MLR), and support vector machine (SVM)).	Developing and comparing three machine learning algorithms to estimate BPs using PPG only, revealed that the regression of the tree algorithm was the best approach with overall acceptable accuracy to ISO standard for BP device validation. Regression tree algorithm achieved acceptable measurement accuracy only in the normotensive category, suggesting that future algorithm development for BP estimation should be more specific for different BP categories. Limitations include manual check for PPG quality, limited clinical variables and limited databases available.

ABR: Adaptive Boosting Regression; AH: Arterial Hypertension; AI: Artificial Intelligence; ANN: Artificial Neural Network; AUC: Area Under the Curve; BMI: Body Mass Index; BN: Bayesian Network classifier; BP: Blood Pressure; CVD: Cardiovascular Disease; DBP: Diastolic Blood Pressure; DNN: Deep Neural Network; GBR: Gradient-Boosting Regression; HC: Hip Circumference; LB: LogitBoost; LRM: Logistic Regression Model; LSTM: Long Short-Term Memory; LWB: Locally Weighted Naive Bayes; ML:

Machine Learning; NLP: Natural Language Processing; PPG: Photoplethysmography; RFR: Random Forest regression; RTF: Random Tree Forest SBP: Systolic Blood Pressure; SNP: Single-Nucleotide Polymorphism; SVM: Support Vector Machine; WC: Waist Circumference; WHR: Waist Hip Ratio; XGBoost: Extreme Gradient Boosting.

Table S2. Major contributions of AI in AH prediction and diagnosis.

Reference (y)	Application/Aim	Methods/Population	Results/Limitations
Ye et al. (2020) PMID: 32339929 [5]	Prediction of models to identify optimal AH treatment pathways in each patient.	245499 hypertensive patients were extracted and processed. Recurrent ANN, including long short-term memory (LSTM) and bi-directional LSTM, was formed into 3 stages: the training set, the validation set, and the test set with a ratio of 6:2:2 respectively of study population.	The recurrent ANN model, especially LSTM model were able to predict the individual probability of achieving optimal BP control. Limitations include lack of information on compliance, post-treatment follow-up, and exclusion of incomplete patient dataset.
LaFreniere et al. (2016) DOI: 10.1109/SSCI.2016.7849886 [6]	Utilized ANN to predict the presence of AH in susceptible individuals.	The hypertensive group consisted of 185371 patients and control group consisted of 193656 patients seeking medical aid for other reasons. Eleven risk factors of interest were used as input data to elaborate, process, and learn the ANN model.	ANN predicted AH with about 82% accuracy. Limitations include lack of information on lab results and complete patient dataset.
Ye et al. (2018) PMID: 29382633 [7]	AI model to predict the incidence of essential AH.	Retrospective (N=823,627, calendar year 2013) and prospective (N=680,810, calendar year 2014) cohorts were formed. ML algorithm was adopted in the process of feature selection and model building, to assign a risk prediction model of essential hypertension.	ML approach achieved a predictive precision of 0.917 in the retrospective cohort and of 0.870 in the prospective cohort. Limitations include lack of information on comorbidities and lifestyle patients data-set.
Kanegae et al (2020) PMID: 31816148 [8]	Using machine learning techniques to develop and validate a new risk prediction model for new-onset hypertension	2005-2016 health checkup data from 18 258 individuals, at the time of hypertension diagnosis [Year (0)] and in the two previous annual visits [Year (−1) and Year (−2)]. Data were entered into models based on machine learning methods (XGBoost and ensemble) or traditional statistical methods (logistic regression). Data was randomly split into a derivation set (75%, n = 13 694) used for model construction and development, and a validation set (25%, n = 4564) used to test performance of the derived models.	The best predictor in the XGBoost model was systolic blood pressure during cardio-ankle vascular index measurement at Year (−1). Area under the receiver operator characteristic curve values in the validation cohort were 0.877, 0.881, and 0.859 for the XGBoost, ensemble, and logistic regression models, respectively. Limitations include method of BP measurement and limited health information.
Golino et al (2014) PMID: 24669313 [9]	Prediction of increased blood pressure by body mass index (BMI), waist (WC) and hip circumference (HC), and waist hip ratio (WHR) using a machine learning technique named classification tree	Data were collected from 400 college students (56.3% women) from 16 to 63 years old. Fifteen trees were calculated in the training group for each sex, using different numbers and combinations of predictors.	This model had a sensitivity of 72% and specificity of 86.25% in the training set and, respectively, 58.38% and 69.70% in the test set. Classification tree analysis outperformed traditional logistic regression. Limitations include use of convenience sample, limited sex variable, and limited health information.
Huang et al. (2010) PMID: 20505678 [10]	Examining risk factors for AH and to develop a prediction model to estimate AH risk for rural residents over the age of 35 years.	Cross-sectional survey of 3054 rural community residents (N=3054). Participants were divided into two groups: a training set (N1=2438) and a validation set (N2=616). The predictors of AH risk were identified from the	The ANN model (area under the curve (AUC)=0.900+/-0.014) proved better than the LRM (AUC=0.732+/-0.026) in terms of evaluating the AH risk because

		training set using logistic regression analysis. The predictive models used were logistic regression model (LRM) and artificial neural network (ANN).	with a larger area under the ROC curve. Limitations include limited geographical and ethnical patient samples, and single BP measurement.
Held et al (2016) PMID: 27980626 [11]	The use of machine learning approach for predicting disease risk by genotypes to be able to incorporate gene expression data and rare variants.	2 different versions of the approach (radial and linear support vector machines) to simulated data from Genetic Analysis Workshop 19 and compare performance to logistic regression.	Method performance was not different across the 3 methods, although the linear support vector machine tended to show small gains in predictive ability relative to a radial support vector machine and logistic regression. The linear support vector machine showed more robust performance to the inclusion of additional genes. Limitations include model used, small sample size, and only use of intragenic SNPs.
Li et al. (2017) PMID: 27895194 [12]	The use of machine-learning algorithm to predict new hypertension genes.	Using the protein interaction network data 222 genes from the HPO (Online Predicted Human Interaction Database Hypertension) data set and 744 genes from the T-HOD (Text-Mined Hypertension, Obesity and Diabetes Candidate Gene Database) data set were covered by the OPHID network and used for network analysis.	Machine-learning algorithm predicted one hundred and seventy-seven new hypertension genes with a posterior probability >0.9. Evidence supporting 17 of the predictions has been found. Limitations include the evaluation of only positively expressed genes.
Maxwell et al. (2017) PMID: 29297288 [13]	Multi-label classification of data for Intelligent health risk prediction models	Physical examination records of 110,300 anonymous patients were used to predict diabetes, hypertension, fatty liver, a combination of these three chronic diseases, and the absence of disease (8 classes in total). The dataset was split into training (90%) and testing (10%) sub-datasets. Ten-fold cross validation was used to evaluate prediction accuracy with metrics such as precision, recall, and F-score.	Deep Neural Networks (DNN), a DL architecture, when applied to multi-label classification of chronic diseases, produced accuracy that was comparable to that of common methods such as Support Vector Machines. Limitations include lack of expert knowledge for network learning.
Sakr et al. (2018) PMID: 29668729 [14]	Performance of different machine learning techniques on predicting the individuals at risk of developing hypertension, and who are likely to benefit most from interventions, using the cardiorespiratory fitness data.	Information of 23,095 patients who underwent clinician-referred exercise treadmill stress testing at Henry Ford Health Systems between 1991 and 2009 and had a complete 10-year follow-up. The variables of the dataset include information on vital signs, diagnosis, and clinical laboratory measurements. Six machine learning techniques were investigated: LogitBoost (LB), Bayesian Network classifier (BN), Locally Weighted Naive Bayes (LWB), Artificial Neural Network (ANN), Support Vector Machine (SVM) and Random Tree Forest (RTF).	The RTF model has shown the best performance (AUC = 0.93) and outperformed all other machine learning techniques examined in this study. Limitations include limited geographical and ethnical patient samples.
Huan et al. (2015) PMID: 25882670 [15]	Blood pressure associated molecular mechanisms by integrating blood pressure GWAS with whole blood mRNA expression profiles	Blood pressure transcriptomic signatures at the single-gene and the co-expression network module levels were identified. Four co-expression modules were identified as potentially causal based on genetic inference	Blood pressure related genes validated were SH2B3. Genes predicted to be regulated by SH2B3 due to an exaggerated pressor response to angiotensin II

	in 3,679 individuals, using network approaches.	because expression- related SNPs for their corresponding genes demonstrated enrichment for blood pressure GWAS signals. Genes from the four modules were further projected onto predefined molecular interaction networks, revealing key drivers.	infusion. Limitations include single-gene-based analyses and complex network analyses for regulatory gene and gene-gene interaction evaluation.

ABR: Adaptive Boosting Regression; AH: Arterial Hypertension; AI: Artificial Intelligence; ANN: Artificial Neural Network; AUC: Area Under the Curve; BMI: Body Mass Index; BN: Bayesian Network classifier; BP: Blood Pressure; CVD: Cardiovascular Disease; DBP: Diastolic Blood Pressure; DNN: Deep Neural Network; GBR: Gradient-Boosting Regression; HC: Hip Circumference; LB: LogitBoost; LRM: Logistic Regression Model; LSTM: Long Short-Term Memory; LWB: Locally Weighted Naive Bayes; ML: Machine Learning; NLP: Natural Language Processing; PPG: Photoplethysmography; RFR: Random Forest regression; RTF: Random Tree Forest SBP: Systolic Blood Pressure; SNP: Single-Nucleotide Polymorphism; SVM: Support Vector Machine; WC: Waist Circumference; WHR: Waist Hip Ratio; XGBoost: Extreme Gradient Boosting.

Table S3. Major contributions of AI in AH treatment.

Reference (y)	Application/Aim	Methods/Population	Results/Limitations
Koren et al. (2018) PMID: 29721321 [16]	Utility of machine learning of big data in gaining insight into the treatment of hypertension. To identify concomitant drugs not considered to have antihypertensive activity,	Machine learning techniques such as decision trees and neural networks, to identify determinants that contribute to the success of hypertension drug treatment on a large set of patients.	Beta blockers, PPIs and statins from machine learning of a large and diverse set of big data holds potential promise for Hypertension. Limitations resides in LM techniques data interpretation.
Duan et al. (2019) PMID: 30857410 [17]	Develop models that estimate individual treatment effects for patients that may most benefit from intensive blood pressure therapy in proportion to baseline risk. Machine learning approach designed to predict heterogeneous treatment effects—the X-learner meta-algorithm—is equivalent to a conventional logistic regression approach.	Comparison between conventional logistic regression to the X-learner approach for prediction of 3-year CVD event risk reduction from intensive versus standard blood pressure treatment, using individual participant data from the SPRINT (N=9361) and ACCORD-BP (N=4733) trials. Each model incorporated 17 covariates, an indicator for treatment arm, and interaction terms between covariates and treatment.	Predictions for individual treatment effects from trial data revealed that patients may experience ARRs not simply proportional to baseline CVD risk. Machine learning methods may improve discrimination and calibration of individualized treatment effect estimates from clinical trial data. Limitations resides in difficulty of analyses interpretation.
Ye et al. (2020) PMID: 32339929 [5]	Test the feasibility of using deep learning predictive models to identify optimal hypertension treatment pathways for individual patients, based on empirical data available from an electronic health record database.	Data on 245,499 unique patients who were initially diagnosed with essential hypertension and received anti-hypertensive treatment using recurrent neural networks (RNN), including long short-term memory (LSTM) and bi-directional LSTM, to create risk-adapted models to predict the probability of reaching the BP control targets associated with different BP treatment regimens.	LSTM models achieved high accuracy when predicting individual probability of reaching BP goals on different treatments for both systolic and diastolic blood pressure, F1-scores were 0.928, 0.960, and 0.913, respectively. Limitations include use of convenience sample and lack of long-term monitoring.

ABR: Adaptive Boosting Regression; AH: Arterial Hypertension; AI: Artificial Intelligence; ANN: Artificial Neural Network; AUC: Area Under the Curve; BMI: Body Mass Index; BN: Bayesian Network classifier; BP: Blood Pressure; CVD: Cardiovascular Disease; DBP: Diastolic Blood Pressure; DNN: Deep Neural Network; GBR: Gradient-Boosting Regression; HC: Hip Circumference; LB: LogitBoost; LRM: Logistic Regression Model; LSTM: Long Short-Term Memory; LWB: Locally Weighted Naive Bayes; ML: Machine Learning; NLP: Natural Language Processing; PPG: Photoplethysmography; RFR: Random Forest regression; RTF: Random Tree Forest SBP: Systolic Blood Pressure;

SNP: Single-Nucleotide Polymorphism; SVM: Support Vector Machine; WC: Waist Circumference; WHR: Waist Hip Ratio; XGBoost: Extreme Gradient Boosting.

Table S4. Major contributions of AI in AH outcome.

Reference (y)	Application/Aim	Methods/Population	Results/Limitations
Chang et al. (2019) PMID: 31703364 [18]	A prediction method for outcomes based on physical examination indicators of hypertension patients.	By first extracting key features from the patients' physical examination indicators and secondly by using key features extracted from the first step to predict the patients' outcomes. A model combining recursive feature elimination with a cross-validation method and classification algorithm. Once recursive feature elimination algorithm to rank the importance of all features, and then extract the optimal features subset using cross-validation was done, the four classification algorithms (support vector machine (SVM), C4.5 decision tree, random forest (RF), and extreme gradient boosting (XGBoost)) to accurately predict patient outcomes by using their optimal features subset were used.	C4.5, RF, and XGBoost can achieve very good prediction results with a small number of features, and the classifier after recursive feature elimination with cross-validation feature selection has better prediction performance. The XGBoost showed the best prediction performance, and its accuracy, F1, and area under receiver operating characteristic curve (AUC) values are 94.36%, 0.875, and 0.927, respectively, using the optimal features subset. Limitations include use of simple deletion method, small dataset, and lack of advanced algorithms.
Wu et al. (2020) PMID: 32172622 [19]	Feasibility of an ML approach for predicting outcomes in young patients with hypertension.	Baseline clinical data and a composite end point—comprising all-cause death, acute myocardial infarction, coronary artery revascularization, new-onset heart failure, new-onset atrial fibrillation/atrial flutter, sustained ventricular tachycardia/ventricular fibrillation, peripheral artery revascularization, new-onset stroke, end-stage renal disease—were evaluated in 508 young patients with hypertension who had been treated at a tertiary hospital. Construction of the ML model, which consisted of recursive feature elimination, extreme gradient boosting, and 10-fold cross-validation, was performed at the 33-month follow-up evaluation, and the model's performance was compared with that of the Cox regression and recalibrated Framingham Risk Score models.	An 11-variable combination was considered most valuable for predicting outcomes using the ML approach. The C statistic for identifying patients with composite end points was 0.757 (95% CI, 0.660–0.854) for the ML model, whereas for Cox regression model and the recalibrated Framingham Risk Score model it was 0.723 (95% CI, 0.636–0.810) and 0.529 (95% CI, 0.403–0.655). Limitations include small sample size from single tertiary referral centre with short follow-up period.

ABR: Adaptive Boosting Regression; AH: Arterial Hypertension; AI: Artificial Intelligence; ANN: Artificial Neural Network; AUC: Area Under the Curve; BMI: Body Mass Index; BN: Bayesian Network classifier; BP: Blood Pressure; CVD: Cardiovascular Disease; DBP: Diastolic Blood Pressure; DNN: Deep Neural Network; GBR: Gradient-Boosting Regression; HC: Hip Circumference; LB: LogitBoost; LRM: Logistic Regression Model; LSTM: Long Short-Term Memory; LWB: Locally Weighted Naive Bayes; ML: Machine Learning; NLP: Natural Language Processing; PPG: Photoplethysmography; RFR: Random Forest regression; RTF: Random Tree Forest SBP: Systolic Blood Pressure; SNP: Single-Nucleotide Polymorphism; SVM: Support Vector Machine; WC: Waist Circumference; WHR: Waist Hip Ratio; XGBoost: Extreme Gradient Boosting.

Table S5. Limitations of applying ML in cardiovascular research.

ML limitations in CV research
• Time and resources
• Patient's data privacy
• Bias (eg. sample bias, unconscious human bias)
• Need for high quality data training
• Not unanimously recognized CV risk factors

Reference

1. Huang, K.H.; Tan, F.; Wang, T.D.; Yang, Y.J. A Highly Sensitive Pressure-Sensing Array for Blood Pressure Estimation Assisted by Machine-Learning Techniques. Sensors (Basel) **2019**, 19, doi:10.3390/s19040848.
2. Chowdhury, M.H.; Shuzan, M.N.I.; Chowdhury, M.E.H.; Mahbub, Z.B.; Uddin, M.M.; Khandakar, A.; Reaz, M.B.I. Estimating Blood Pressure from the Photoplethysmogram Signal and Demographic Features Using Machine Learning Techniques. Sensors (Basel) **2020**, 20, doi:10.3390/s20113127.
3. Chen, S.; Ji, Z.; Wu, H.; Xu, Y. A Non-Invasive Continuous Blood Pressure Estimation Approach Based on Machine Learning. Sensors (Basel) **2019**, 19, doi:10.3390/s19112585.
4. Khalid, S.G.; Zhang, J.; Chen, F.; Zheng, D. Blood Pressure Estimation Using Photoplethysmography Only: Comparison between Different Machine Learning Approaches. J Healthc Eng **2018**, 2018, 1548647, doi:10.1155/2018/1548647.
5. Ye, X.; Zeng, Q.T.; Facelli, J.C.; Brixner, D.I.; Conway, M.; Bray, B.E. Predicting Optimal Hypertension Treatment Pathways Using Recurrent Neural Networks. Int J Med Inform **2020**, 139, 104122, doi:10.1016/j.ijmedinf.2020.104122.
6. LaFreniere, D.; Zulkernine, F.; Barber, D.; Martin, K. Using machine learning to predict hypertension from a clinical dataset. In Proceedings of the 2016 IEEE Symposium Series on Computational Intelligence (SSCI), Athens, Greece, 06-09 December 2016, 2016.
7. Ye, C.; Fu, T.; Hao, S.; Zhang, Y.; Wang, O.; Jin, B.; Xia, M.; Liu, M.; Zhou, X.; Wu, Q.; et al. Prediction of Incident Hypertension Within the Next Year: Prospective Study Using Statewide Electronic Health Records and Machine Learning. J Med Internet Res **2018**, 20, e22, doi:10.2196/jmir.9268.
8. Kanegae, H.; Suzuki, K.; Fukatani, K.; Ito, T.; Harada, N.; Kario, K. Highly precise risk prediction model for new-onset hypertension using artificial intelligence techniques. J Clin Hypertens (Greenwich) **2020**, 22, 445-450, doi:10.1111/jch.13759.
9. Golino, H.F.; Amaral, L.S.; Duarte, S.F.; Gomes, C.M.; Soares Tde, J.; Dos Reis, L.A.; Santos, J. Predicting increased blood pressure using machine learning. J Obes **2014**, 2014, 637635, doi:10.1155/2014/637635.
10. Huang, S.; Xu, Y.; Yue, L.; Wei, S.; Liu, L.; Gan, X.; Zhou, S.; Nie, S. Evaluating the risk of hypertension using an artificial neural network method in rural residents over the age of 35 years in a Chinese area. Hypertens Res **2010**, 33, 722-726, doi:10.1038/hr.2010.73.
11. Held, E.; Cape, J.; Tintle, N. Comparing machine learning and logistic regression methods for predicting hypertension using a combination of gene expression and next-generation sequencing data. BMC Proc **2016**, 10, 141-145, doi:10.1186/s12919-016-0020-2.
12. Li, Y.H.; Zhang, G.G.; Wang, N. Systematic Characterization and Prediction of Human Hypertension Genes. Hypertension **2017**, 69, 349-355, doi:10.1161/HYPERTENSIONAHA.116.08573.
13. Maxwell, A.; Li, R.; Yang, B.; Weng, H.; Ou, A.; Hong, H.; Zhou, Z.; Gong, P.; Zhang, C. Deep learning architectures for multi-label classification of intelligent health risk prediction. BMC Bioinformatics **2017**, 18, 523, doi:10.1186/s12859-017-1898-z.
14. Sakr, S.; Elshawi, R.; Ahmed, A.; Qureshi, W.T.; Brawner, C.; Keteyian, S.; Blaha, M.J.; Al-Mallah, M.H. Using machine learning on cardiorespiratory fitness data for predicting hypertension: The Henry Ford ExercIse Testing (FIT) Project. PLoS One **2018**, 13, e0195344, doi:10.1371/journal.pone.0195344.
15. Huan, T.; Meng, Q.; Saleh, M.A.; Norlander, A.E.; Joehanes, R.; Zhu, J.; Chen, B.H.; Zhang, B.; Johnson, A.D.; Ying, S.; et al. Integrative network analysis reveals molecular mechanisms of blood pressure regulation. Mol Syst Biol **2015**, 11, 799, doi:10.15252/msb.20145399.
16. Koren, G.; Nordon, G.; Radinsky, K.; Shalev, V. Machine learning of big data in gaining insight into successful treatment of hypertension. Pharmacol Res Perspect **2018**, 6, e00396, doi:10.1002/prp2.396.
17. Duan, T.; Rajpurkar, P.; Laird, D.; Ng, A.Y.; Basu, S. Clinical Value of Predicting Individual Treatment Effects for Intensive Blood Pressure Therapy. Circ Cardiovasc Qual Outcomes **2019**, 12, e005010, doi:10.1161/CIRCOUTCOMES.118.005010.
18. Chang, W.; Liu, Y.; Xiao, Y.; Yuan, X.; Xu, X.; Zhang, S.; Zhou, S. A Machine-Learning-Based Prediction Method for Hypertension Outcomes Based on Medical Data. Diagnostics (Basel) **2019**, 9, doi:10.3390/diagnostics9040178.
19. Wu, X.; Yuan, X.; Wang, W.; Liu, K.; Qin, Y.; Sun, X.; Ma, W.; Zou, Y.; Zhang, H.; Zhou, X.; et al. Value of a Machine Learning Approach for Predicting Clinical Outcomes in Young Patients With Hypertension. Hypertension **2020**, 75, 1271-1278, doi:10.1161/HYPERTENSIONAHA.119.13404.
